# Retraction note: Cyto-histopathological and outcome features of the prepuce squamous cell carcinoma of a mixed breed dog

**DOI:** 10.1186/s13000-016-0577-0

**Published:** 2016-11-02

**Authors:** Javad Yaghoobi Yeganeh Manesh, Radmehr Shafiee, Ali Mohammad Bahrami, Mehdi Pourzaer, Maryam Pourzaer, Behnam Pedram, Javad Javanbakht, Aram Mokarizadeh, Farshid Khadivar

**Affiliations:** 1 Gradute of Islamic Azad University of Shahrekord, Faculty of Veterinary Medicine, Shahrekord University, Shahrekord, Iran; 2 Graduate, Faculty of Veterinary Medicine, Tehran University, Tehran, Iran; 3 Faculty of Para Veterinary Medicine, Ilam University, Ilam, Iran; 4 Graduate Student, Faculty of Veterinary Science, Karaj Branch, Islamic Azad University, Alborz, Iran; 5 Department of Biology, Faculty of Basic Science, Damghan Branch, Islamic Azad University, Damghan, Iran; 6 Department of Pathobiology, Susangerd Branch, Islamic Azad University, Susangerd, Iran; 7 Department of Pathology, Faculty of Veterinary Medicine, Tehran University, Tehran, Iran; 8 Department of Immunology, Faculty of Medicine, Kurdistan University of Medical Sciences, Sanandaj, Iran

## Retraction

The Editor-in-Chief and Publisher have retracted this article [1] because the scientific integrity of the content cannot be guaranteed. An investigation by the Publisher found it to be one of a group of articles we have identified as showing evidence suggestive of attempts to subvert the peer review and publication system to inappropriately obtain or allocate authorship. This article showed evidence of plagiarism (most notably from the articles cited [2–7]) and peer review and authorship manipulation.
